# Giant pseudoaneurysm following percutaneous coronary intervention: a case report

**DOI:** 10.3389/fcvm.2025.1701880

**Published:** 2025-12-05

**Authors:** Lin Tian, Zhiyuan Wang, Kun Liu, Yaliang Tong, Yuquan He, Xiangdong Li, Guohui Liu

**Affiliations:** Department of Cardiology, China-Japan Union Hospital of Jilin University, Jilin University, Changchun, Jilin, China

**Keywords:** coronary artery pseudoaneurysm, chronic coronary artery occlusion, percutaneous coronary intervention, covered stent, coronary artery rupture, cardiac tamponade

## Abstract

A coronary pseudoaneurysm (CPA) is defined as a rupture of the coronary artery wall where blood is encapsulated by surrounding pericardial tissue, thrombus, or fibrous tissue, forming a fluid-filled sac lacking normal vascular walls. While pseudoaneurysms can develop spontaneously, they most commonly occur following coronary intervention procedures, such as percutaneous coronary intervention. Causes include excessive balloon or stent usage, high-pressure balloon dilation, and arterial wall damage from coronary atherosclerotic resection or laser angioplasty. Although asymptomatic in many cases, a ruptured CPA can lead to life-threatening acute cardiac tamponade. Notably, postoperative mortality in some PCI patients may result from both malignant arrhythmias and recurrent myocardial infarction, with CPA rupture being a contributing factor. This case report describes a patient who developed a massive pseudoaneurysm 1 year after undergoing chronic coronary occlusion lesion recanalization. Through surgical review, we analyze the etiology, discuss subsequent interventions, and highlight clinical lessons to provide guidance for managing such complex cases.

## 1 Case profiles

A 46-year-old male patient was admitted to the Department of Cardiovascular Medicine, China–Japan Union Hospital of Jilin University, on December 20, 2024. Four years earlier, the patient first experienced angina pectoris during physical activity. Subsequently, he underwent coronary angiography at a local hospital, which revealed three-vessel disease. Stents were implanted in the left anterior descending artery, circumflex artery, and right coronary artery. The patient was discharged after his symptoms were relieved and he started regular medication. One year prior to current admission, the patient was admitted to our hospital due to recurrent angina pectoris. Repeat angiography showed that the left coronary stent was normal, but the proximal right coronary artery and the stent were completely occluded. After the occlusion of the right coronary artery was opened, the patient received drug-eluting balloon dilation within the stent and stent implantation in the proximal right coronary artery. His symptoms improved, and he was discharged. Two weeks prior to current admission, the patient once again experienced angina pectoris after physical activity. Oral medication did not provide significant relief, so he was admitted to our department with the chief complaint of “intermittent dull pain in the precordial area after intermittent physical activity for 4 years, aggravated for 2 weeks.” The patient had no history of chronic infectious diseases, hypertension, diabetes, or other related diseases.

The patient had no history of surgery, trauma, allergy, or exposure to epidemic areas. However, he has an 11-year history of smoking approximately 10 cigarettes per day and has not quit to date.

**Figure 1:**
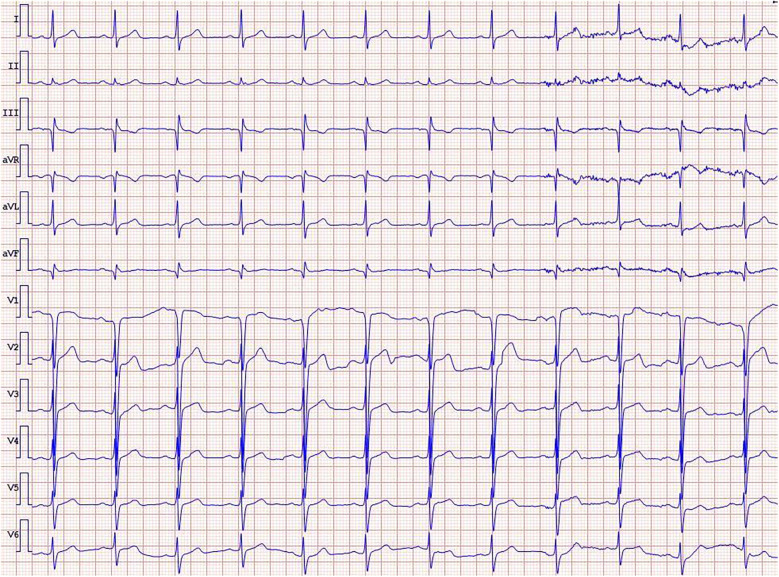
ECG on admission.

**Figure 2:**
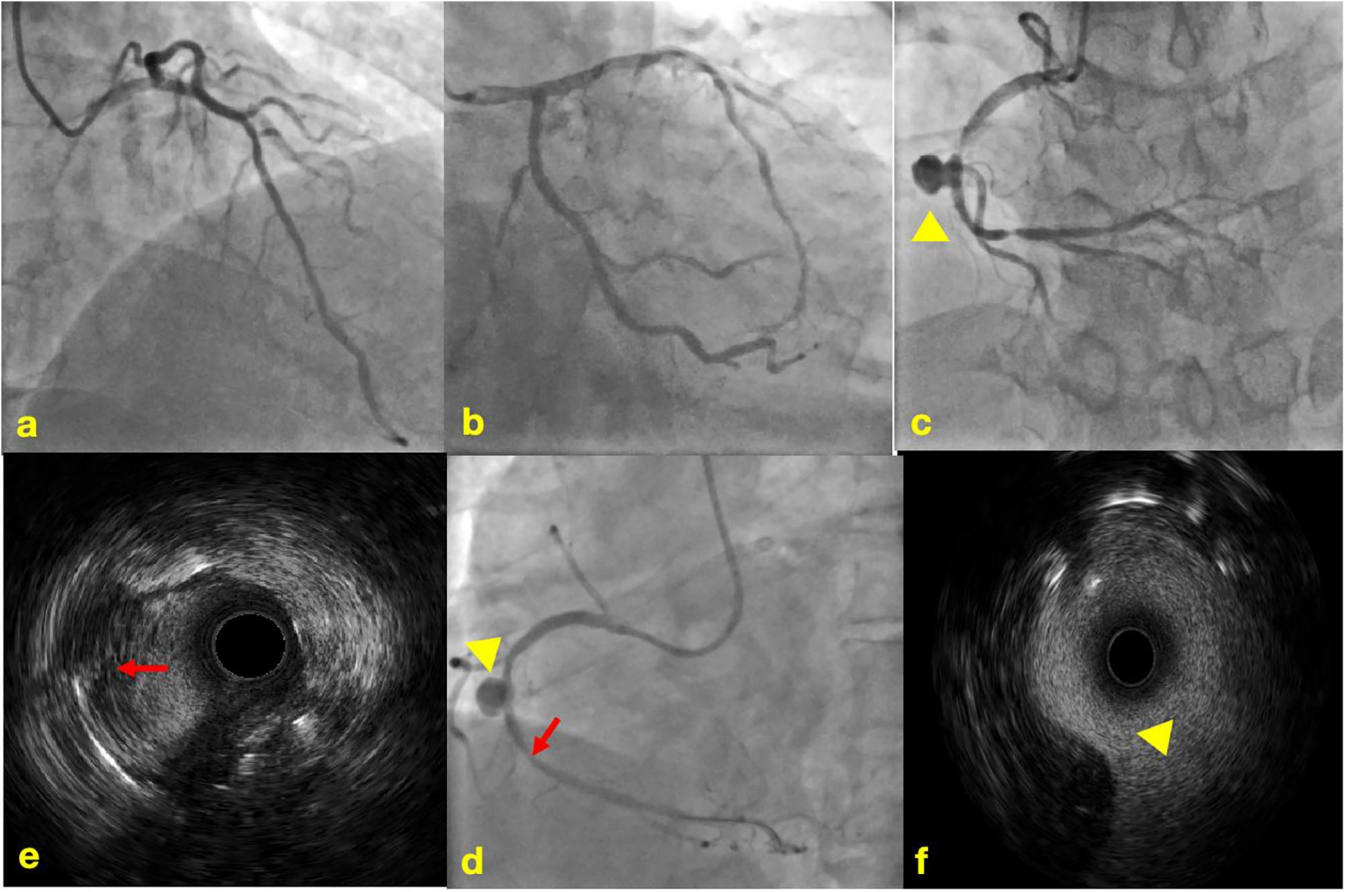
Coronary angiography and intravascular ultrasound. **(a)** Normal imaging of the LAD stent; **(b)** normal imaging of the LCX stent; **(c)** imaging of in-stent restenosis and coronary artery aneurysm in the RCA; **(d)** imaging of post-balloon dilation in the RCA; **(e)** IVUS image of newly formed atherosclerotic plaque; **(f)** IVUS image of coronary artery aneurysm, lacking the normal three-layer structure of arteries. 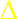 The yellow triangle shows coronary artery aneurysm. 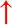 The red arrow shows newly formed atherosclerotic plaque.

**Figure 3:**
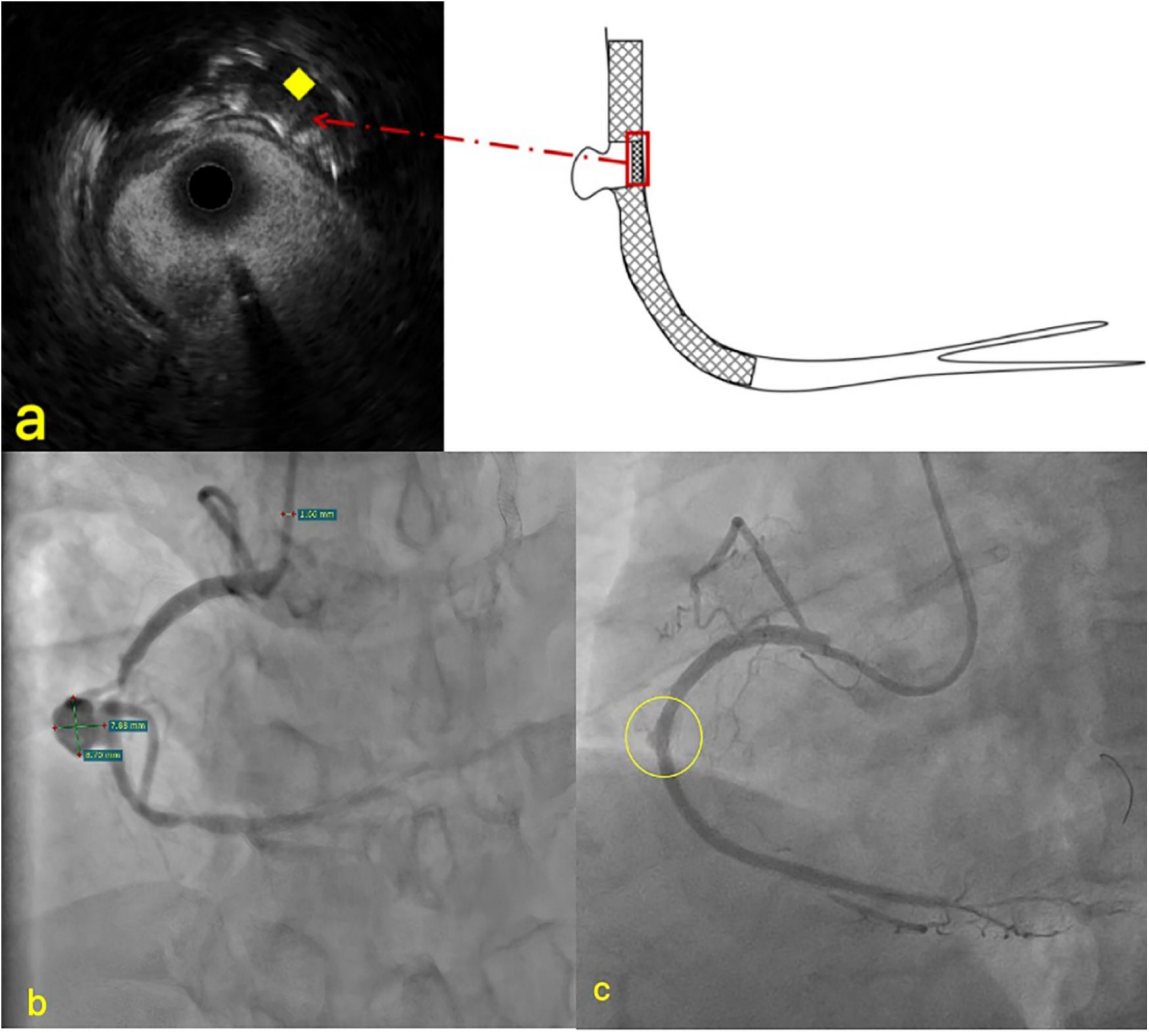
Imaging of PCI and IUVS for pseudoaneurysms. **(a)** The stent at the pseudoaneurysm site was compressed to the opposite side; **(b)** the specific size measurement diagram of pseudoaneurysm; **(c)** Contrast agent leakage. ♦ The yellow rhombus indicates the compressed stent in IVUS.

Physical examination revealed no significant abnormalities. Laboratory test results (Table 1) indicated poor glycemic control, dyslipidemia, and mild liver dysfunction. The initial ECG (Figure 1) showed R-wave progression in anterior leads and the presence of pathological q waves in lead III and aVF. Cardiac ultrasound revealed the following measurements: left ventricular diameter 48.5 mm, interventricular septal thickness 10.4 mm, left ventricular posterior wall thickness 10.2 mm. The echocardiogram revealed regional wall motion abnormalities with a preserved global left ventricular ejection fraction of 61.5%. Despite optimized pharmacotherapy, recurrent angina symptoms persisted over the preceding 2 weeks, which were not resolved. Therefore, a coronary angiography was performed (Figures 2a–d), which revealed normal stent conditions in the left anterior descending artery and circumflex artery, while an aneurysmal bulge was observed in the right mid-coronary artery with proximal and distal restenosis. We conducted intravascular ultrasound (IVUS) of the right coronary artery (Figures 2e,f), identifying new atherosclerotic plaques as the cause of restenosis. The proximal lumen bulge area exhibited incomplete vascular wall structure, leading to a diagnosis of pseudoaneurysm. Coronary angiography revealed that the maximum diameter of the pseudoaneurysm was approximately 8.70 mm (Figure 3b).

**Table 1:** Laboratory test results.

	FBG	TG	LDL-C	LP(a)	ALT	AST	TNI	BNP
Numeric value	7.11 mmol/L	4.57 mmol/L	3.05 mmol/L	65.88 mg/dL	75.93 IU/L	56.96 IU/L	Negative	Negative

FBG, fasting blood-glucose; TG, triglyceride; LDL-C, low-density lipoprotein cholesterol; LP(a), lipoprotein-a; ALT, alanine aminotransferase; AST, aspartate aminotransferase; TnI, troponin I; BNP, B-type natriuretic peptide.

**Table 2:** Summary of CPA-related treatment in 5 years.

Ref.	Article title	Age and gender	treat	final result
(14)	Spontaneous closure of a coronary artery bypass graft pseudoaneurysm embedded in a mediastinal hematoma	Male, 73 years old	Spontaneous closure	Discharge
(15)	Spontaneous resolution of a pseudoaneurysm at the anastomosis site of coronary artery bypass graft	Male, 66 years old	Spontaneous closure	No recurrence within 1 year
(16)	Spontaneous regression of post-traumatic left anterior descending coronary pseudoaneurysm	Male, 27 years old	Expectant treatment	After 3 years of follow-up, coronary CTA showed spontaneous regression of LAD pseudotrauma
(17)	Right coronary artery button pseudoaneurysm after the modified Bentall procedure	Male, 30 years old	Surgical operation	He was discharged on the fifth day after surgery
(18)	Surgical management of a rare right coronary artery fistula with a giant pseudoaneurysm compressing the pulmonary vein in a young patient	Female, 11 years old	Surgical operation	No recurrence during follow-up
(19)	Coronary artery pseudoaneurysm 13 years after stent implantation	Male, 78 years old	Covers and frames	Four months after surgery, CT was reviewed without recurrence
(11)	Percutaneous coil embolization of a post-traumatic left anterior descending coronary artery pseudoaneurysm: a case report	Male, 78 years old	Spring coil	Asymptomatic recurrence after 1 month and death after 3 months
(20)	Repair of a right coronary artery rupture with perforated right ventricle following spontaneous pseudoaneurysm: a case report	Male, 68 years old	Covershield	Patient died
(21)	A giant right coronary artery pseudoaneurysm successfully managed with a covered stent	Female, 80 years old	Covers and frames	No symptoms were maintained during the 6-month follow-up
(8)	Treatment of coronary pseudoaneurysm detected after percutaneous coronary intervention for chronic total occlusion	Male, 40 years old	Covers and frames	No CPA-like manifestations were observed during 5-month follow-up
(22)	Progression and interventional therapy of a coronary pseudoaneurysm: a case report	Male, 55 years old	Covers and frames	No symptoms were maintained during the 6-month follow-up
(23)	Coil embolization for ruptured coronary pseudoaneurysm causing haemopericardium: a case report	Male, 15 years old	Spring coil	He was discharged 12 days after surgery
(24)	Successful percutaneous intervention of contained coronary artery rupture: the role of intracoronary imaging	Male, 66 years old	Covers and frames	Discharge
(25)	Management of a giant dual-chamber pseudoaneurysm of the proximal left anterior descending artery	Male, 73 years old	Covers and frames	No obvious abnormalities were found in the 3-month follow-up

## 2 Treatment decision and course

In most post-PCI cases, the formation of coronary pseudoaneurysms is typically attributed to guidewire-induced vascular wall damage and localized coronary perforation during high-pressure balloon dilation. To investigate the specific etiology of this patient's coronary pseudoaneurysm (CPA), we conducted a thorough review of the surgical procedure conducted 1 year earlier. Coronary angiography at that time revealed proximal occlusion of the right coronary artery. During the operation, PILOT200 and Gaia 3 guidewires were successfully employed to recanalize the right coronary CTO, without performing IVUS. Subsequently, NC balloons were repeatedly expanded to address residual stenosis within the stent, followed by stent implantation in the proximal segment and pharmacoballoon therapy at the site of secondary stenosis. Final imaging demonstrated minor contrast leakage in the mid-coronary artery, which disappeared after intermittent pressure application during balloon inflation. Postoperative echocardiography showed no significant pericardial effusion. The conclusion was drawn that the right coronary pseudoaneurysm originated from local vessel wall rupture during interventional therapy conducted 1 year previously. However, given the moderate balloon expansion pressure and minimal vascular calcification, why did vessel wall rupture occur? Through detailed analysis of this IVUS imaging, absence of stent trabeculae was identified at the aneurysm site. Retrograde angiographic evidence also showed partial compression of the stent toward one side (Figure 3). These findings indicate that, during CTO recanalization, the guidewire partially traversed beneath the stent mesh in the proximal right coronary artery, located subintimally, leading to vessel rupture following balloon dilation.

**Figure 4:**
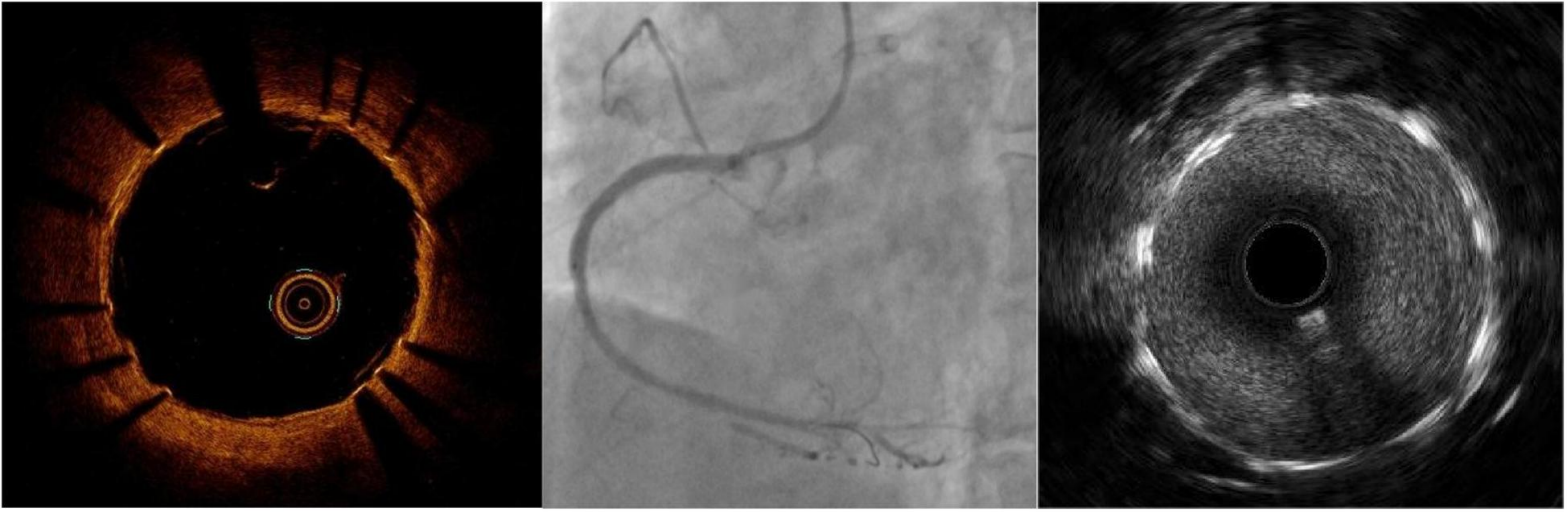
The RCA images after implanting a covered stent in OCT, CAG, and IVUS.

Given the patient's large aneurysm with a wide neck, which could rupture at any moment, posing an imminent risk of cardiac tamponade, we urgently considered two treatment options: coil embolization or stent placement. The coil approach was ruled out due to uncertainty regarding the wall integrity of the pseudotumor and the risk of rupture, leading us to select a stent graft. With no suitable stent available and time being critical, we fabricated a 4.0 × 15 mm stent graft using a 3M-coated drug-eluting stent. We cut the 3M film into long rectangles, with the width shorter than the length of the support. The ends were positioned approximately 1 mm away from the edge of the support. While keeping the film stationary, the support was gently rotated, wrapping the film for one and a half turns, avoiding excessive tension. The excess 3M film was cut off, resulting in a successfully covered support. For fabrication of the covered stent, it is important to maintain a certain tension during the winding of the membrane, while avoiding excessive tension. When transporting the stent, it was moved gently. If resistance was encountered, it was retracted and delivered once again after thorough pretreatment. A Runthrough guidewire was used to advance the covered stent to the pseudoaneurysm site within the middle segment of the right coronary artery stent. The stent was expanded and released at 14 atmospheres for 10 s. Then, a 4.0*15 mm NC balloon was utilized at 20 atmospheres for optimization. Reexamination with intravascular ultrasound (IVUS) showed a strip-like high-echo shadow in the middle segment lumen. Further OCT examination (Figure 4) revealed that the high-echo bright band was a fibrous plaque dissection within the stent. Subsequently, we performed drug-coated balloon therapy at the site of restenosis within the stent.

To prevent thrombosis, the patient received intensive dual antiplatelet therapy (DAPT; aspirin 100 mg daily, ticagrelor 90 mg twice daily) following surgery. Concurrently, lipid-lowering treatment was initiated with atorvastatin 20 mg daily combined with PCSK9 inhibitors (ilosunimab 140 mg every 2 weeks), along instructions to quit smoking. During the 1-year follow-up after discharge, the patient reported no chest pain symptoms, other discomforts, or adverse cardiovascular events.

## 3 Discussion

Aortic aneurysms are classified as true aneurysms or pseudoaneurysms based on arterial wall integrity. True aneurysms retain complete endarterial, media, and adventitious membrane structures, whereas coronary artery pseudoaneurysms develop due to wall defects, lacking at least one arterial layer and being encapsulated by surrounding tissues (1). Coronary pseudoaneurysms are extremely rare, typically occurring after catheter-based percutaneous coronary intervention (PCI) procedures. These typically develop from invasive coronary dissection or perforation, causing vessel wall damage without involvement of the adventitious membrane, necessitating heightened awareness of iatrogenic CPA risks (2–4). In complex chronic coronary occlusion (CTO) cases (particularly those following prior interventional failure), PCI interventions may require guidewire modification and escalation. While these techniques improve PCI success rates in complex CTO cases, they may compromise the elastic components of the media layer, promoting pseudoaneurysm dilation and hematoma formation. Notably, high-pressure dilation after stent implantation can thin and remodel vessel walls, exacerbating CPA development. Preoperative coronary CT angiography and intraoperative intravascular ultrasound have been shown to enhance PCI success rates for anatomically complex CTO cases (including vascular anomalies, occlusions, missing proximal remnants, severe tortuosity, or long-segment occlusions with unclear etiology), while reducing CPA occurrence. In cases of catheter wall rupture with minor contrast agent leakage during surgery, a method of intermittent balloon dilation at a 1:1 ratio to the lumen diameter can be employed. Simultaneously, active use of intracavitary imaging is suggested. For patients temporarily stabilized through balloon dilation and occlusion therapy, in addition to close monitoring of vital signs during hospitalization, increased frequency of CTA follow-up examinations after discharge will enable early detection of pseudoaneurysm formation.

Coronary artery pseudoaneurysms pose a significant management challenge due to their unpredictable natural history and potential for life-threatening complications, such as rupture, thrombosis, or distal embolism. These fragile lesions, composed solely of thrombus and fibrous tissue, are extremely vulnerable to rupture under sustained arterial pressure. Rupture can rapidly cause blood to flood the pericardial cavity, triggering acute cardiac tamponade, which may lead to sudden shock or death. Moreover, the chaotic blood flow within pseudoaneurysms promotes thrombus formation. When these clots detach, they may obstruct distal coronary arteries through circulation, causing acute myocardial infarction. Progressive enlargement of the aneurysm can compress adjacent coronary arteries, resulting in angina or ischemia. In rare cases, massive aneurysms may compress ventricles or conduction systems, leading to heart failure or arrhythmias. The gold standard for diagnosing coronary pseudoaneurysms is coronary angiography, while coronary CTA and echocardiography also provide diagnostic value, with intravascular ultrasound aiding differentiation between true aneurysms and pseudoaneurysms (5). This particular case was confirmed through integration of patient history, coronary angiography, and intravascular ultrasound. Current literature indicates that untreated PCI-related CPA may lead to adverse outcomes (6), primarily due to cardiac tamponade caused by CPA rupture, ultimately resulting in patient death.

Currently, there is no universally recommended standard treatment for PCI-related CPA. The main options include conservative observation, membrane-coated stent implantation, and coil embolization. The choice depends on the size of the aneurysm, its location, neck width, whether it continues to expand, and hemodynamic effects. Previous studies have demonstrated (7) that endovascular treatment with membrane-coated stents is particularly suitable for single-coronary-branch aneurysms with normal left ventricular function. In this case, we performed implantation of a membrane-coated stent using OCT to identify CPA openings and determine optimal stent placement. The stent featured a polymer membrane (typically ePTFE) over its metallic framework, which helps prevent plaque prolapse, restores vascular wall integrity, reduces blood flow impact on plaques, and immediately physically isolates vascular tears following implantation to block blood entry into the aneurysm cavity. This significantly improves immediate outcomes, making it one of the preferred methods for acute perforations and subsequent pseudoaneurysms. Such stents prove especially effective for wide-necked aneurysms, particularly those with unfavorable morphologies such as a high dome-to-neck ratio or located in tortuous parent arteries, where conventional coil embolization may be challenging due to the risk of coil prolapse. In addition, while occluding tears, this type of stent maintains original blood flow to avoid myocardial ischemia. Recent studies have reported (8) acceptable safety profiles with relatively low cardiac adverse event rates. However, certain drawbacks persist. First, the stent's rigidity makes it difficult to navigate through twisted or calcified vessels, requiring high surgical skill. Second, when implanted near critical branch vessels, the stent may “seal” these branches, potentially causing myocardial infarction. The primary concern stems from the slower endothelialization process of covered stents, which increases the risk of intimal hyperplasia and leads to a more frequent long-term restenosis rate compared to drug-eluting stents. The presence of the covered material also increases the risk of thrombosis, necessitating intensified DAPT. The current standard protocol combines aspirin with a P2Y12 inhibitor (e.g., clopidogrel or ticagrelor). For covered stents—particularly when treating complex conditions like pseudoaneurysms—the recommended DAPT duration typically spans 6–12 months. Patients at high risk of thrombosis (including those with diabetes, renal impairment, complex lesions, or long stents) may require extended DAPT therapy (up to 12 months). In cases where potent DAPT is accompanied by elevated bleeding risks (such as a history of hemorrhage, advanced age, or oral anticoagulant therapy), downgrading or shortening the treatment course after 6 months can be considered. Therefore, determining the optimal duration requires careful balancing between thrombotic and hemorrhagic risks.

In practice, many catheterization labs lack pre-packaged covered stents, necessitating on-site fabrication by interventional physicians (9). The use of covered stents in the treatment of coronary artery perforation has been widely recognized as an effective approach in clinical practice (10). The covered stent creates an airtight pipeline at the perforation site, isolating the breach from the blood flow and promoting local thrombosis and endothelial coverage, thereby achieving permanent closure. This is similar to the principle used in surgical procedures where artificial vascular patches are employed to repair vascular defects.

We cut 3M™ Tegaderm™ transparent film dressings to the required length (the width of the film is shorter than the length of the stent, leaving approximately 1 mm from each end to the edge of the stent) and wrapped them around the drug-eluting stent surface with 1.5 complete turns. During the wrapping process, the film was kept stationary while the stent was gently rotated with a certain tension. We were careful to avoid excessive tension and cut off the excess 3M film. The ends of the covered stent were then fixed with a very small dose of medical-grade cyanoacrylate glue. This glue, widely used in clinical practice for skin adhesion and vascular embolization, has the characteristics of rapid polymerization and good tissue compatibility. When making the self-made covered stent, DES was selected as the platform based on its availability and its potential inhibitory effect on edge restenosis. Although bare-metal stents offer lower cost and can be considered an option, their long-term effects need to be comprehensively evaluated in specific clinical situations. The covered design used for this patient is a permanent implant, aiming to provide long-lasting mechanical support and occlusion for the vascular wall. Its long-term biocompatibility and safety have been confirmed through extensive clinical applications. While custom-made coronary covered stents can effectively prevent leakage from arterial perforation, their poor endothelialization increases risks of thrombosis and restenosis. To mitigate these issues, intensified anticoagulation and antiplatelet therapy should be prioritized over routine use of makeshift stents. Although coronary angiography is the primary method for assessing the success of covered stent implantation in this emergency settings, subsequent intravascular imaging examinations (such as OCT) can provide more precise evaluation of the stent's adherence to the vessel wall and its interaction with the vessel wall, thereby offering more information relating to long-term prognosis. In the present case, post-release OCT imaging confirmed aneurysm lumen closure and stent patency. The patient received 12-month intensive dual antiplatelet therapy and lipid-lowering treatment following surgery, with no adverse events reported during follow-up.

Spring coil embolization involves delivering metal coils through microcatheters into the space of a pseudoaneurysm, filling the cavity to induce thrombus formation and occlusion. This technique selectively targets aneurysms while preserving the patency of the supplying artery. For aneurysms adjacent to critical branch vessels, this technique avoids coverage of the branches. Successful embolization allows for lower antiplatelet therapy intensity compared to covered stents. In addition, spring coil therapy serves as a salvage option when covered stent implantation fails. However, the procedure is complex and requires strict aneurysm neck specifications, making it most suitable for “gourd-shaped” aneurysms with large bodies and narrow necks. For wide-necked aneurysms, coils may detach into the supplying artery, causing distal embolism. Post-treatment thrombus organization reduces the volume of compressed coils, potentially leading to recurrence (11). In this case, due to uncertainty about the toughness of the pseudoaneurysm wall, we opted against coil therapy to prevent rupture.

A literature review revealed that a small subset of untreated coronary perforation (CAPA) patients demonstrated positive clinical outcomes during follow-up, with two cases achieving spontaneous healing. Other patients underwent coronary artery bypass grafting (CABG), pseudoaneurysm resection, or interventional therapy. Researchers have proposed that using covered stents or PTFE-coated stents for occlusion may be more effective in CAPA cases associated with PCI (8). With continuous advancements in cardiac interventional techniques, the incidence of CPA has increased; however, data regarding rupture rates and long-term prognoses remain inconclusive (12, 13). Table 2 provides a summary of treatment approaches for CPA over the past 5 years.

## 4 Conclusion

Pseudoaneurysms, a rare complication of PCI, carry an extremely high risk of rupture and pose life-threatening consequences. Prevention is therefore paramount, taking precedence over treatment. During complex PCI procedures, comprehensive endovascular imaging should be performed to minimize potential risks. Treatment plans for pseudoaneurysms should be customized based on the characteristics of the lesion, including the use of covered stents, coil embolization, or CABG. Once covered stent placement is confirmed, postoperative DAPT must be intensified, with close monitoring of stent patency and bleeding status. During PCI, if vascular wall leakage is detected, CT angiography (CTA) frequency should be appropriately increased to enable early detection of pseudoaneurysm formation.

## Data Availability

The original contributions presented in the study are included in the article/Supplementary Material; further inquiries can be directed to the corresponding author.
