# Correction: Giant pseudoaneurysm following percutaneous coronary intervention: a case report

**DOI:** 10.3389/fcvm.2025.1764239

**Published:** 2025-12-19

**Authors:** Frontiers Production Office

**Affiliations:** Frontiers Media SA, Lausanne, Switzerland

**Keywords:** coronary artery pseudoaneurysm, chronic coronary artery occlusion, percutaneous coronary intervention, covered stent, coronary artery rupture, cardiac tamponade

Author Xiangdong Li was erroneously not assigned as a co-corresponding author. The correct corresponding authors are Xiangdong Li and Guohui Liu.

The original version of this article has been updated.

